# Temperature-dependent sex-reversal by a *transformer-2* gene-edited mutation in the spotted wing drosophila, *Drosophila suzukii*

**DOI:** 10.1038/s41598-017-12405-4

**Published:** 2017-09-28

**Authors:** Jianwei Li, Alfred M. Handler

**Affiliations:** USDA/ARS, Center for Medical, Agricultural and Veterinary Entomology, 1700 SW 23rd Drive, Gainesville, FL 32608 USA

## Abstract

Female to male sex reversal was achieved in an emerging agricultural insect pest, *Drosophila suzukii*, by creating a temperature-sensitive point mutation in the sex-determination gene, *transformer-2* (*tra-2*), using CRISPR/Cas9 (clustered regularly interspaced palindromic repeats/CRISPR-associated) homology-directed repair gene-editing. *Ds-tra-2* ^*ts2*^ mutants developed as normal fertile XX and XY adults at permissive temperatures below 20 °C, but at higher restrictive temperatures (26 to 29 °C) chromosomal XX females developed as sterile intersexuals with a predominant male phenotype, while XY males developed with normal morphology, but were sterile. The temperature-dependent function of the Ds-TRA-2^ts2^ protein was also evident by the up- and down-regulation of female-specific *Ds-Yolk protein 1* (*Ds-Yp1*) gene expression by temperature shifts during adulthood. This study confirmed the temperature-dependent function of a gene-edited mutation and provides a new method for the more general creation of conditional mutations for functional genomic analysis in insects, and other organisms. Furthermore, it provides a temperature-dependent system for creating sterile male populations useful for enhancing the efficacy of biologically-based programs, such as the sterile insect technique (SIT), to control *D. suzukii* and other insect pest species of agricultural and medical importance.

## Introduction

The role of the *transformer-2* (*tra-2*) sex determination gene in *Drosophila melanogaster*, and the temporal specificity of the hierarchal sex-determination gene pathway was elucidated in part by the selection of two *tra-2* temperature-sensitive mutant alleles induced by chemical mutagenesis, which allowed its function and downstream effects to be controlled by ambient temperature^1,2^. Two missense point mutations, resulting in Ala151Val (GCC > GTC) for *tra-2* ^*ts1*^ and Pro181Ser (CCA > TCA) for *tra-2* ^*ts2*^, apparently result in temperature-labile conformational changes in the polypeptide at the elevated temperature of 29 °C that eliminate function, while normal function is maintained at 16 °C. The importance of these amino acids is also reflected in their conservation in *tra-2* cognates found in other drosophilids, tephritids, a lepidopteran and two hymenopteran species^3^, that include the Ala161 and Pro191 peptides in the spotted winged drosophila, *D. suzukii* ^4^.

Notably, the ability to manipulate TRA-2 protein function during development provided the initial insight that its function, and thus, that of the sex-determination gene hierarchy, was required continuously for somatic female-specific differentiation, and in the transient absence of TRA-2, the default state of male differentiation would result^1,2^. The notion that this sex-specific control extended past metamorphosis, and was required throughout adulthood after completion of morphological differentiation, was supported by experiments using female-specific *yolk protein* (*Yp*) gene expression as a sex-specific biochemical and molecular marker during adulthood. By subjecting XX; *tra-2* ^*ts*^ flies to shifts in ambient temperature it was discovered that *Yp* gene expression and protein synthesis could be diminished at 29 °C and re-initiated when shifted back to 16 °C. This showed that *tra-2* function acted continuously in adult females to maintain Yp synthesis, and thus, female-specific processes throughout adulthood^5^.

In addition to the fundamental knowledge of how sex-determination genes control sex-specific development and behavior, the *tra-2* ^*ts2*^ studies, in particular, also presented an ideal strategy for generating a sterile male population that could be of use to the sterile insect technique (SIT)^6^, a highly effective biologically-based means of controlling the population size of a variety of insect pest species^7–9^. A cornerstone of SIT is the mass release of sterile males that, in sufficient numbers, outcompete wild males in the field for female mating thereby rendering them non-reproductive. Limitations to these programs has generally been the need for sexing in early development to eliminate the high cost of rearing females and the need to sterilize and release them with males in the absence of sexing, and the debilitating somatic effects of irradiation used for sterilization^10^.

The *tra-2* ^*ts2*^ mutation effectively addresses both of these limitations as demonstrated in *D. melanogaster*, where homozygous male and female *tra-2* ^*ts2*^ mutants reared at 16 °C develop normally and are fertile, but when reared at 29 °C, chromosomal XX females develop as sterile phenotypic (or pseudo) males that exhibit courtship behavior (observed in XX; *tra-2* ^*ts1*^/*tra-2* ^*def*^)^11^. XY chromosomal males develop normally at the restrictive temperature, but are sterile due to requisite TRA-2 function in the germ-line^12,13^. Thus, a *tra-2* ^*ts2*^ mutant strain could provide a normal breeding population at the permissive temperature, whose progeny reared at the restrictive temperature would all develop as sterile males for release. Two important benefits of this strategy are that: i) no other treatment other than elevated temperature would be required for both sexing and male sterility in drosophilid pests, such as *D. suzukii* (though other non-drosophilid species may require an independent sterility system for XY; *tra-2* ^*ts2*^ males), and ii) a smaller breeding population may be sufficient to generate the same number of phenotypic sterile males required for release, substantially increasing efficiency and cost effectiveness.

The ability to incorporate this strategy into insect pest species having an orthologous *tra-2* gene, however, has been hindered by the need to generate the same mutation for a mass reared strain in the absence of the genetic tools available for *D. melanogaster*, including visible selectable markers and balancer chromosomes. Germ-line transformation could be used to introduce an *in vitro* mutagenized *tra-2* ^*ts*^ allele, though targeted mutagenesis would be required to eliminate function of the native wild type alleles that would complement the mutant loss of function. These restrictions can be largely overcome by gene-editing techniques that allow a gene conversion or replacement of the native wild type *tra-2* allele for a mutated *tra-2* ^*ts*^ allele, in conjunction with incorporating a dominant fluorescent protein visible marker for ease of selection and rearing^14^. To demonstrate the feasibility of this approach in an insect pest species, we have created the *Ds-tra-2* ^*ts2*^ mutation by CRISPR/Cas9 editing in the spotted wing drosophila, *D. suzukii*. While this species is not an ideal subject for *tra-2* ^*ts*^-based sex-reversal, since it is not highly viable at optimal restrictive temperatures^15^, temperature-dependent phenotypes of morphological and biochemical sex reversal, and male and female sterility were demonstrated. Thus, this model for temperature-dependent sex reversal and sterility provides proof of concept for further testing in other species subject to control by SIT, and as a method for generating conditional alleles for functional genomic analysis in non-model species.

## Results

### Creation of the *Ds-tra-2*^*ts2*^ mutation

**Figure 1:**
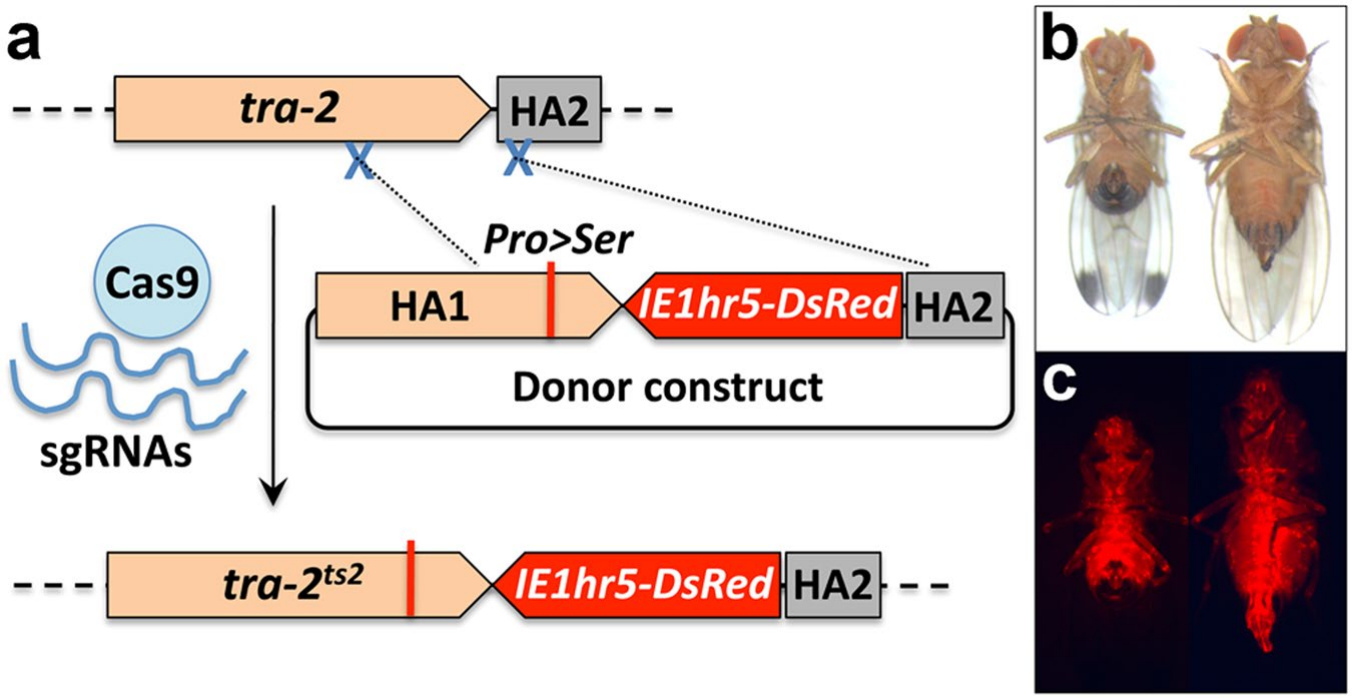
Strategy for generating the *Ds-tra2* ^*ts2*^ mutation and *IE1hr5-DsRed* marker insertion via CRISPR/Cas9 homology-directed repair. (**a**) Schematic (not to scale) showing the wild type *Ds-tra-2* gene and downstream HA2 (homologous arm 2) sequence with expected recombination sites (X) for the Cas9/sgRNA complexes (top). Dotted lines from these sites extend to the donor construct recombination sites that include the HA1 *Ds-tra-2* sequence mutagenized *in vitro* to create a Pro191Ser substitution (vertical red line) resulting in the mutated Ds-TRA2^ts2^ temperature sensitive protein, in addition to the *IE1hr5-DsRed* marker gene and HA2 sequence. The recombined donor construct resulting in the genomic insertion of the *Ds-tra2* ^*ts2*^ mutation and marker gene is shown below. (**b**) Ventral view of a homozygous *Ds-tra2* ^*ts2*^ adult XY male (left) and XX female (right) reared at 16 °C under brightfield, and (**c**) DsRed epifluorescence illumination. See Methods for further details.

CRISPR/Cas9 gene editing was performed using a homology-directed repair (HDR) strategy (Fig. 1a). Two sgRNAs were used as scissors, and a donor construct provided a template including the putative *Ds-tra-2* ^*ts2*^ point mutation, the *IE1hr5-DsRed* marker gene, and two homologous arms upstream and downstream of the cutting site (~1 kb each). One sgRNA targeted *Ds-tra-2* at a position 449 bp 5′ upstream of the mutation site and the other targeted the proximal downstream sequence to *Ds-tra-2*, predicted to be the *biogenesis of lysosome-related organelles complex 1 subunit 1* gene (GenBank: XM_017072992). To bias editing towards HDR, the *ligase 4* gene, a component of the non-homologous end joining (NHEJ) repair pathway, was suppressed using dsRNA-mediated RNA interference (RNAi). A mixture of sgRNAs, Cas9 protein, *Ds-ligase-4* dsRNA and donor construct was injected into pre-blastoderm embryos of a *D. suzukii* wild strain, yielding 81 G0 adult flies (36 males and 45 females). Of these, 55 flies were fertile after individual backcrosses to wild type, with four G0 lines yielding G1 progeny expressing whole body red fluorescence, resulting in a germ-line transmission rate of 7.3% (4/55) based on marker expression. From each G0 line, two to five red fluorescent G1 flies (Fig. 1c) were selected to establish independent G1 lines. After being inbred to homozygosity, six lines were analyzed by PCR and sequencing showing that all lines had an insertion of the *IE1hr5-DsRed* marker gene, however, only three lines (F26m1, F26f4 and M36f1, from two G0 lines) carried the correct *Ds-tra-2* ^*ts2*^ (CCT > TCT) mutation while the other three lines could not be confirmed, resulting in a minimum mutation rate of 3.6% (2/55).

### Temperature sensitivity of Ds-TRA-2 protein function based on adult morphology and fertility

**Figure 2:**
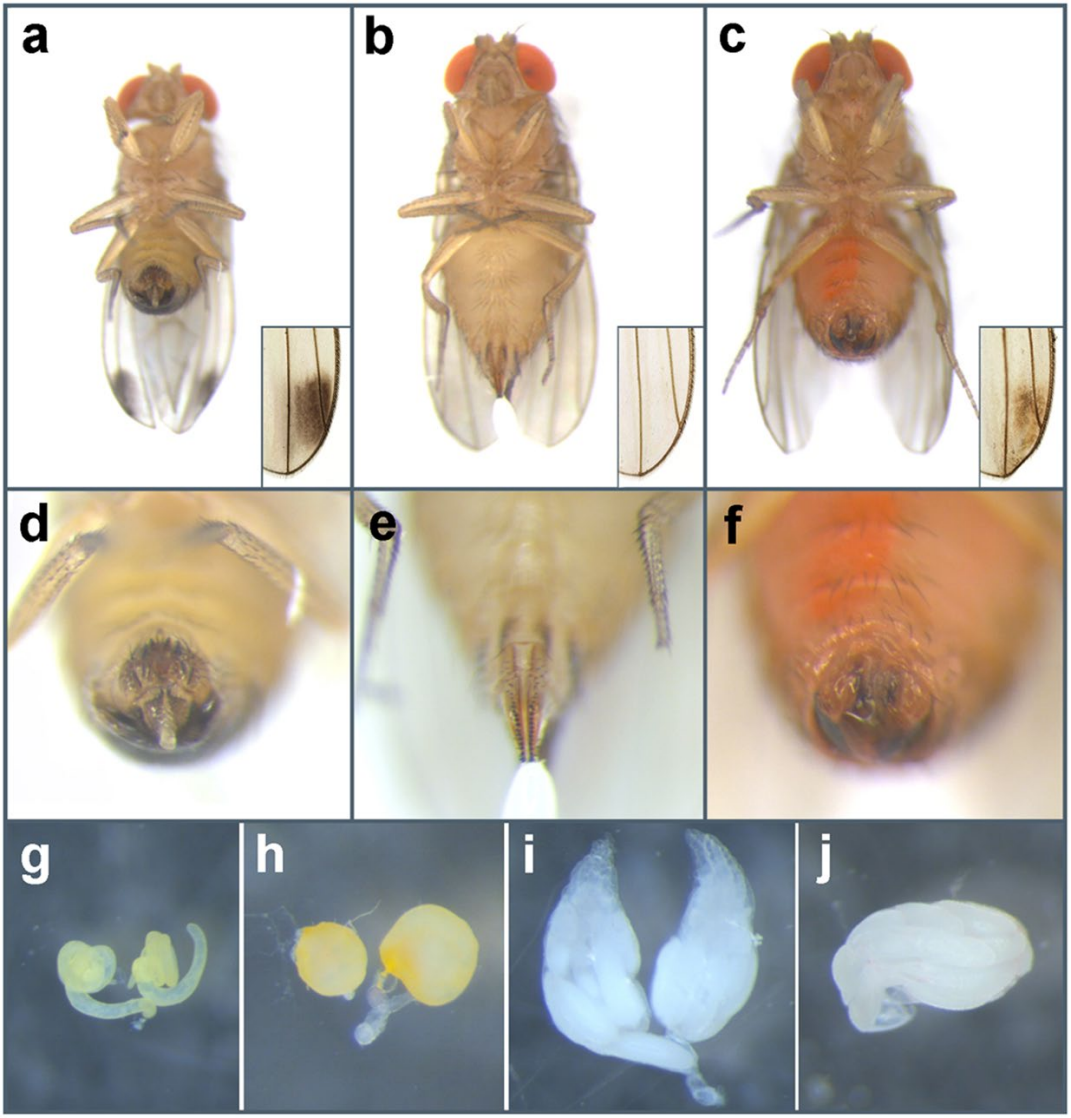
Phenotypic comparison of wild type and mutant flies. Ventral view of an adult *D. suzukii* wild type male (**a**), WT female (**b**), and a homozygous XX; *Ds-tra2* ^*ts2*^ mutant pseudo-male reared at 26 °C (red coloration is from the DsRed marker protein) (**c**). Lower right insets show an enlarged region of the posterior wing where male-specific wing spot pigmentation is present in the WT male (**a**), absent in the WT female (**b**), and is apparent in another XX mutant pseudo-male shifted from 26 °C to 29 °C at pharate adulthood (but absent when maintained at 26 °C) (**c**). An enlarged ventral posterior view of the WT male (**d**), WT female (**e**), and pseudo-male (**f**) showing the genital terminalia apparatus. Gonads dissected from adult flies reared at 26 °C including WT male testes (**g**), XY; *Ds-tra2* ^*ts2*^ male dysmorphic testes (**h**), WT female ovaries (**i**), and an XX; *Ds-tra2* ^*ts2*^ pseudo-male dysmorphic gonad (**j**).

**Figure 3:**
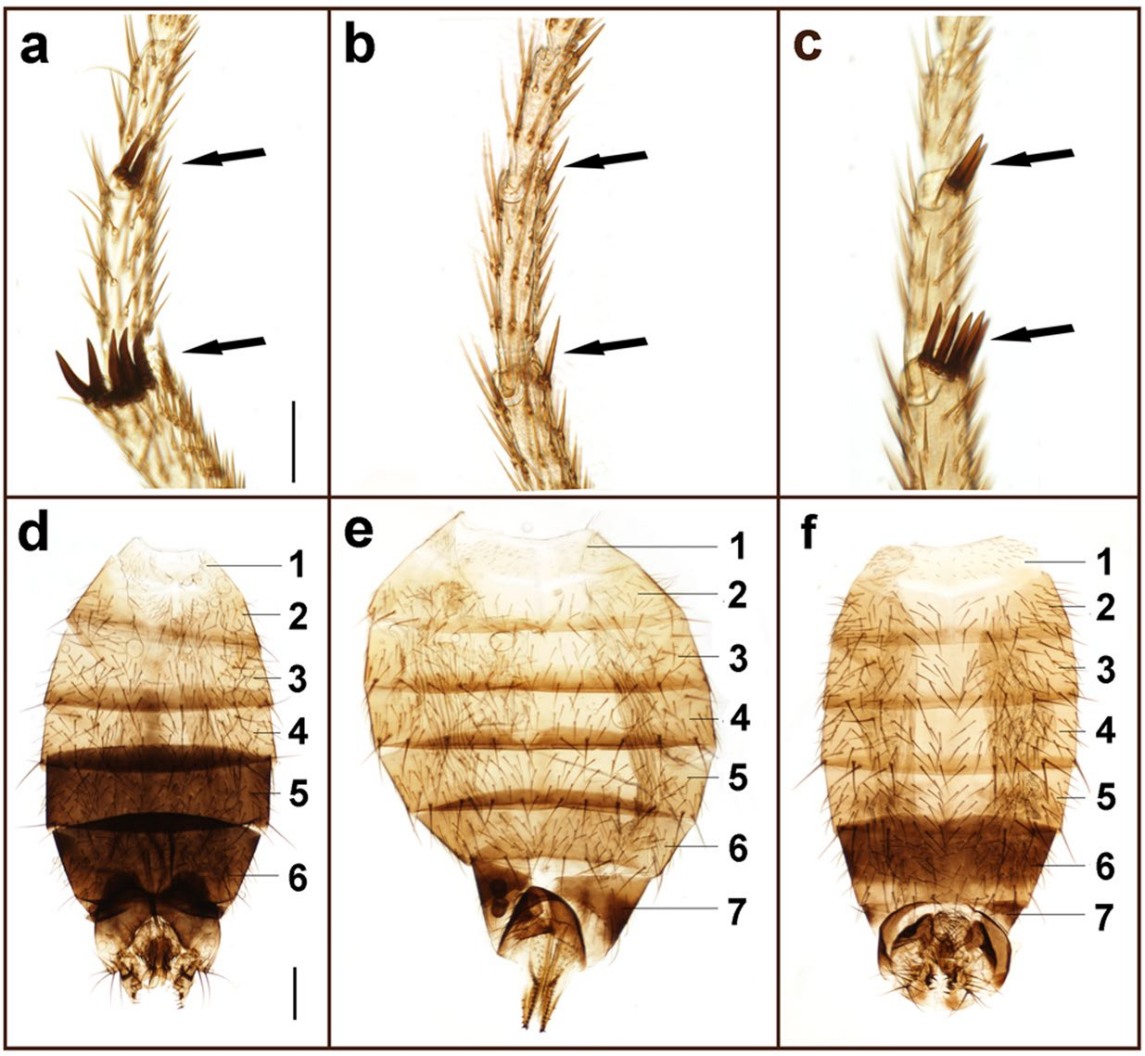
Comparison of sex combs and abdominal pigmentation in wild type and mutant flies. Sex-specific differentiation of forelegs and dorsal abdominal cuticle in a wild type male (**a** and **d**), WT female (**b** and **e**), and an XX; *Ds-tra2* ^*ts2*^ pseudo-male (**c** and **f**) reared at 26 °C. The regions for foreleg sex comb bristle formation (**a**–**c**) are indicated by arrows, and abdominal tergite segments (**d**–**f**) are numbered. Orientation of the forelegs relative to the body are distal at the top and proximal at the bottom of the panels. Scale bars shown for (**a**–**c**) (50 µm) and (**d–f**) (200 µm).

To determine the effect of temperature on the mutated Ds-TRA-2 protein in terms of morphological sexual differentiation in chromosomal females and males, the homozygous mutant line, M36f1, was first reared continuously at the expected permissive temperature of 16 °C, in addition to 20 °C. This resulted in normal development and fertility in all XX females and XY males at both temperatures (Fig. 1b; Table 1). However, when rearing was performed at the expected restrictive temperature of 29 °C, all mutants died during late pupation, as did wild type control flies. Rearing at 27 °C and 28 °C also resulted in inviability for the mutant line and a low survival rate for wild type (1–2%), with low levels of survival (5–10%) for both wild type and mutant flies first observed at 26 °C. At this temperature 80 surviving chromosomal female XX; *Ds-tra-2* ^*ts2*^ homozygotes exhibited an intersexual, though predominantly male (or pseudo-male), phenotype (Fig. 2c and f), but male-specific wing spots were not apparent (Fig. 2c). However, in a separate experiment where XX; *Ds-tra-2* ^*ts2*^ individuals (*n* = 21), were reared at 22 °C and shifted to 29 °C as late pupae, wing spot pigmentation was observed at 3 days after eclosion (Fig. 2c inset; Supplementary Table S1). Compared to mature ovaries from WT females (Fig. 2i), XX; *Ds-tra-2* ^*ts2*^ pseudo-males had a single poorly differentiated gonadal lobe (Fig. 2j) and no internal male reproductive organs, similar to XX; *Dm-tra-2* ^*ts2*^ flies reared at 29 °C^1^. At 26 °C pseudo-males were also sterile with fully differentiated male-specific foreleg sex combs (Fig. 3c) and male-specific pigmentation on tergite 6 (Fig. 3f), while tergite 5 lacked male-specific pigmentation and female-specific tergite 7 was observed. The differing sexual identity of the abdominal tergites, as well as other sex-specific tissues and structures, can most simply be attributed to a variable sensitivity to Ds-TRA-2 function that was diminished in pseudo-males reared at a sub-optimal restrictive temperature.

**Table 1:** Temperature effects on sexual differentiation and fertility in homozygous XX and XY *Ds-tra-2* ^*ts2*^ adults.

Temperature	XX Females			XY Males		
	No.^a^	Intersex^b^	Fertility^c^	No.^a^	Fertility^c^	Dysmorphic testes^d^
16 °C	201	−	25/25	205	25/25	0/15
20 °C	206	−	22/22	210	22/22	0/15
22 °C	153	+	0/61	145	30/30	0/15
24 °C	135	++	0/30	142	20/20	0/12
24.5 °C	84	++	0/35	90	20/20	15/30
25 °C	78	++	0/36	71	20/20	7/16
25.5 °C	52	+++	0/32	54	0/30	15/15
26 °C	80	+++	0/35	75	0/30	20/20

^a^Number of flies examined for sex-specific phenotypes.

^b^(−) denotes normal female morphology; (+) denotes male sex combs and normal female genitalia; (++) denotes sex combs and abnormal genitalia; and (+++) denotes sex combs and male-like genitalia.

^b,c^wild type and F23f1 control females were phenotypically normal and fertile at all listed temperatures (*n* ≥ 20).

^c^number of fertile flies/tested flies; non-fertile females did not oviposit by 14 d after adult eclosion, whereas WT females reared at 20 °C, 22 °C and 24 °C oviposited ≥78 fertile eggs/female (*n* ≥ 39) at each temperature.

^d^number of males with one or two dysmorphic testes/males dissected (see Fig. 2 g and h).

**Figure 4:**
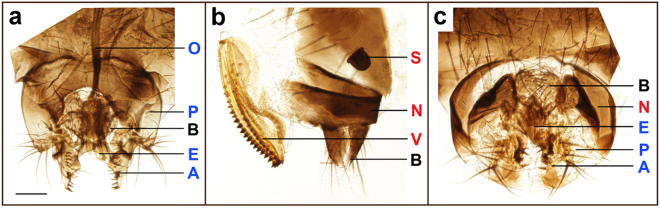
Comparison of genital apparatus in wild type and mutant flies. Genital terminalia of a wild type male (**a**), WT female (**b**) and an XX; *Ds-tra2* ^*ts2*^ pseudo-male (**c**) reared at 26 °C. Abbreviations for indicated structures in each panel: anterior paramere (clasper; A), aedeagus (E), anal plate (B), posterior paramere (P), basal apodeme of the aedeagus (O), vaginal plate (V), female-specific sternite 8 (N), and spermatheca (S). Sex specific structures are labeled in blue for male, red for female, and black for non-sex-specific. Scale bar: 100 µm.

A detailed examination of the external genitalia revealed that pseudo-males exhibited terminalia similar to WT males (Fig. 4), including the anterior paramere (clasper), posterior paramere, aedeagus and anal plates (Fig. 4c). However, the basal apodeme of the aedeagus was not apparent (Fig. 4a) and the female-specific sternite 8 was observed, while the female-specific spermatheca was not observed. At 26 °C, XY; *Ds-tra-2* ^*ts2*^ chromosomal males had normal external morphology, but were sterile consistent with poorly differentiated abnormal testes that had a small number of immotile sperm (*n* = 15, Fig. 2h, Supplementary Video S1), compared to the long spiral testes containing motile sperm found in WT males (Fig. 2g, Supplementary Video S2).

To more precisely determine the temperature sensitivity of Ds-TRA-2 function in the mutant line, adult morphology and fertility were assessed in XY and XX; *Ds-tra-2* ^*ts2*^ adults that developed at rearing temperatures ranging from 16 °C to 26 °C (Table 1). As noted, at the permissive temperatures of 16–20 °C both XX females and XY males were morphologically normal (Fig. 1b) and fertile, but at 22 °C XX females first exhibited male sex combs and were sterile based on a failure to oviposit (*n* = 61), while WT females oviposited ≥78 fertile eggs/female (*n* ≥ 39) when reared at 20–24 °C (Table 1). Increased levels of male morphology occurred with increasing temperature, with nearly complete male morphology at 26 °C. XY males were fertile at 25 °C and below, with some exhibiting dysmorphic testes and sterility at 24–25 °C, that included all males above 25 °C. These results are consistent with Ds-TRA-2 function, or the quantity of functional protein, diminishing just above permissive temperature, with an increasing loss of Ds-TRA-2 activity with rising temperature. The sex-specificity of somatic tissue and germ-line function appeared to be more highly sensitive to loss of Ds-TRA-2 activity in XX females (i.e., onset of sex comb differentiation and sterility) than the influence on testis morphology and germ-line function (i.e., fertility) in XY males where the onset of defects were first detected at a higher temperature.

Complete sterility in XY; *Ds-tra-2* ^*ts2*^ males could be achieved by rearing at 25.5 °C or above, but viability at these temperatures was severely limited. Thus, XY; *Ds-tra-2* ^*ts2*^ males were reared at 22 °C until pharate adulthood or adult eclosion resulting in viability of 80 to 90%, and shifted to 26 °C and/or 29 °C to determine if these late shifts to restrictive temperature would result in sterility (Supplementary Table S1). Sterility was achieved in 90 to 100% of the males, with greater sterility in those that were shifted earlier and to the higher temperature, although this resulted in lower viability.

### Mating behavior of mutant intersexes and pseudo-males

In *D. melanogaster*, temperature sensitive male courtship behavior and mating in XX individuals required a nearly null *Dm-tra-2* genotype that used the stronger *Dm-tra-2* ^*ts1*^ allele over a *Dm-tra-2* deficiency^11^. For XX; *Ds-tra-2* ^*ts2*^ pseudo-males, flies reared at 26 °C (*n* = 20), and shifted to 29 °C for 3d at eclosion from 26 °C (*n* = 15) or as late pupae (*n* = 23) did not attempt courtship or mating with wild type virgin females, while XY; *Ds-tra-2* ^*ts2*^ males reared at 26 °C (*n* = 22) did mate, but were sterile. At 22 °C, XX; *Ds-tra-2* ^*ts2*^ females developed female genital terminalia and did not attempt courtship with WT virgin females (*n* = 29), but were courted by WT and XY; *Ds-tra-2* ^*ts2*^ males who were rejected (*n* = 20).

### Temperature sensitivity of Ds-TRA-2 function based on terminal sex-specific *Yp1* expression during adulthood

Similar to studies on XX; *Dm-tra-2* ^*ts2*^ in *D. melanogaster* ^5^, the influence of Ds-TRA-2 activity on molecular and biochemical aspects of terminal sexual differentiation could be explored by applying temperature shifts to mutant adults. For *D. suzukii* in particular, the ability of adults to survive at 29 °C, after rearing at 26 °C or below, also allowed a more definitive test of the optimal restrictive temperature on Ds-TRA-2^ts2^ protein function. This was achieved by assessing the effect of temperature and temperature shifts on the sex specific expression of the *D. suzukii yolk protein 1* (*Ds*-*Yp1*) gene in *Ds-tra-2* ^*ts2*^ mutants that, in *D. melanogaster*, is normally expressed only in female adult fat body and the ovarian follicular epithelium beginning at adult eclosion^16,17^.

**Figure 5:**
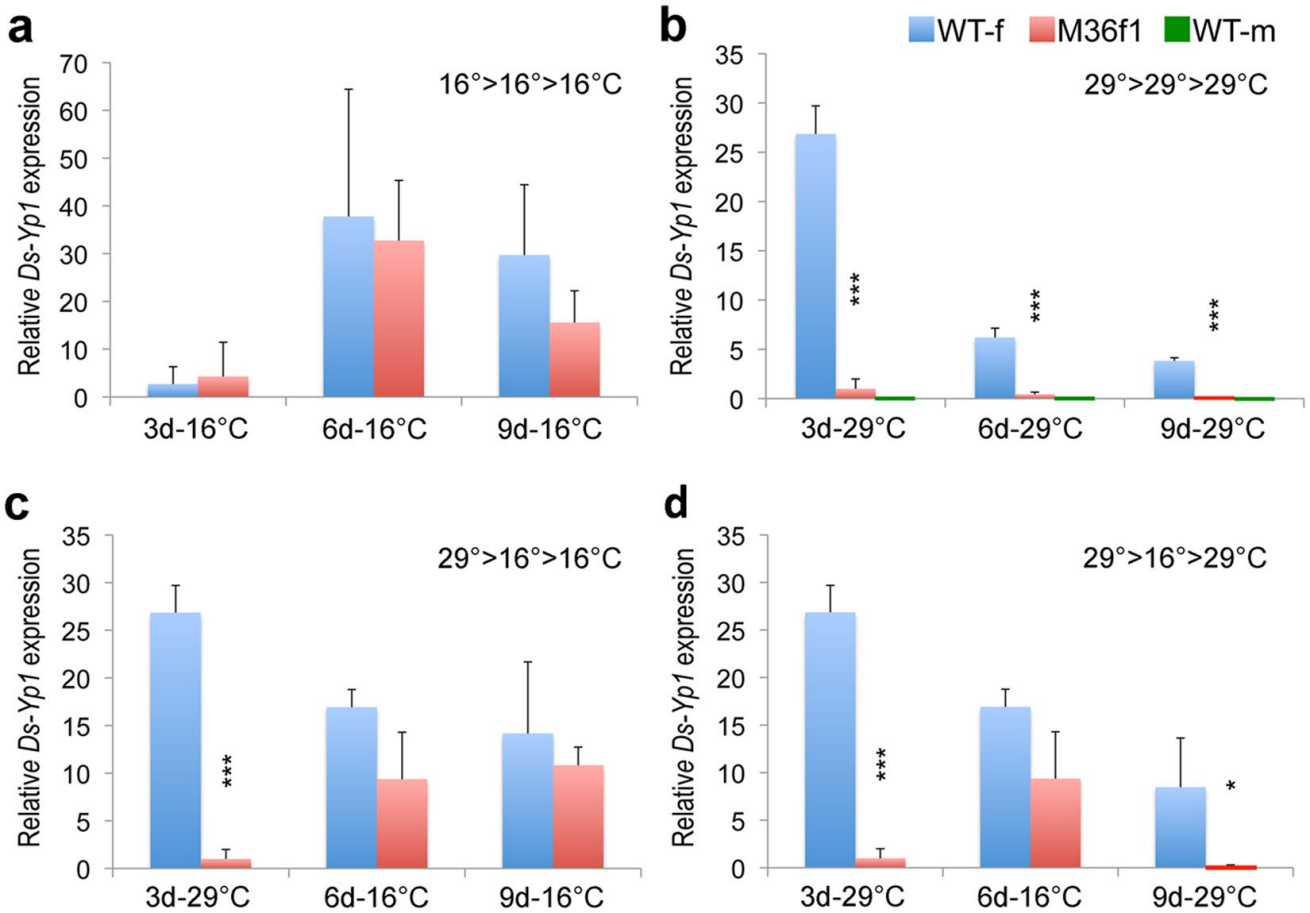
Response of *Ds-Yp1* transcript expression to temperature. *Ds-Yp1* transcript expression (relative to internal *Ds-His3* controls) measured by qPCR from wild type females (WT-f, blue bars), XX; *Ds-tra2* ^*ts2*^ females (M36f1, red bars), and wild type males (WT-m, green bars) in response to indicated temperature and temperature shifts during adulthood. All flies were reared at 16 °C until adult eclosion, and time points indicate days from eclosion and temperatures maintained for the previous three days. Graphs show transcript levels from adults continuously reared at 16 °C (graph **a**) and 29 °C (graph **b**); adults reared from 0–3 days at 29 °C (3d-29 °C; from graph **b**) and then shifted to 16 °C for an additional 3 days (6d-16 °C) and 6 days (9d-16 °C) (graph **c**); or shifted to 29 °C at 6 days (9d-29 °C) (graph **d**; sibling samples from 3d and 6d in graph c shown for comparison). Each bar represents the mean fold change from three biological replicates ± SD relative to sample M36f1 at 3d-29 °C. Asterisks (*) above M36f1 samples indicate *t-test* significance (two-tailed) compared to WT-f at the same time point: ***P < 0.001; *0.01 < *P* < 0.05. Unmarked M36f1 data are not significantly different from WT-f. Transcript levels and *P* values are also presented in Supplementary Table S2.

To measure *Ds-Yp1* transcript levels by qPCR, *Ds-Yp1* was first identified by its similarity to *D. melanogaster Yp1*, having 94.3% amino acid sequence identity (Supplementary Fig. S1), providing primer sites for analysis. Initial qPCR tests for the primers showed that transcript levels were similar in XX WT and F23f1 control females, which were >20-fold higher than transcript in XX; *Ds-tra-2* ^*ts2*^ pseudo-male adults reared at 26 °C (Supplementary Fig. S2). For experimental tests, *Ds-Yp1* transcripts were measured in XX; *Ds-tra-2* ^*ts2*^ (M36f1) phenotypic females and WT females reared and then maintained at 16 °C until 3 d, 6 d, and 9 d after eclosion (Fig. 5a; Supplementary Table S2). Transcript levels were not significantly different between the mutant and WT females at these time points, though transcript increased between days 3 and 6, and then decreased in both WT and XX; *Ds-tra-2* ^*ts2*^ females by day 9, though more so in M36f1 females (*P* > 0.05, two-tailed, no significant difference at all time points; Supplementary Table S2). In contrast, when mutant and WT females reared at 16 °C were shifted to 29 °C at eclosion, after 3 days WT females expressed >27-fold higher levels of *Ds-Yp1* transcript compared to XX; *Ds-tra-2* ^*ts2*^ females (Fig. 5b). Decreasing levels of transcript occurred in both groups at 6 d and 9 d, with negligible transcript detected in mutant females after 9 d at 29 °C. To determine whether the basal transcript level observed in mutant females at 29 °C for 3 days could be reversed to a more normal female phenotype by turning on Ds-TRA-2 function, sibling flies were shifted to 16 °C at 3 d and assayed at days 6 and 9 after eclosion (Fig. 5c). At 6 d the *Ds-Yp1* transcript level in WT females decreased ~40% (presumably due to a slowed metabolism), but increased ~9-fold in XX; *Ds-tra-2* ^*ts2*^ females, with similar levels maintained in both groups at 9 d (*P* > 0.05, two-tailed). However, if females at 6 d at 16 °C were shifted to 29 °C until 9 d, transcript levels decreased by half in WT females, but were barely detectable in the XX; *Ds-tra-2* ^*ts2*^ females, decreasing by >40-fold from day 6 (*P* < 0.05, two-tailed; Fig. 5d).

These results showed that adult XX; *Ds-tra-2* ^*ts2*^ phenotypic females reared at 16 °C exhibit normal female expression of *Ds-Yp1*, but when sibling females were shifted from 16 °C to 29 °C, low diminished transcript levels resulted reaching non-detectable levels, normally expected in XY males, by day 9. Furthermore, *Ds-Yp1* transcript levels increased and decreased according to shifts in temperature to permissive and restrictive conditions, respectively, indicating that the dynamic, and reversible, expression of *Ds-Yp1* during adulthood is continuously regulated by Ds-TRA-2 function. Presumably this function is mediated by the more direct effect of Ds-TRA/Ds-TRA-2 interactions on the female-specific expression of *Ds-dsx* (GenBank: XM_017077018.1)^4^, as demonstrated in *D. melanogaster* ^20^.

## Discussion

Here we report the use of CRISPR/Cas9 gene editing in *D. suzukii* to create a conditional temperature sensitive allele of the *tra-2* sex determination gene, *Ds-tra-2* ^*ts2*^, analogous to the chemically induced *Dm*-*tra-2* ^*ts2*^ missense point mutation in *D. melanogaster* ^1^. This was achieved by comparing amino acid sequence identity for TRA-2 in both species (Supplementary Fig. S3), and identifying the common peptides that are substituted in *Dm*-*tra-2* ^*ts1*^ and *Dm*-*tra-2* ^*ts2*^ ^1,18^. Since *Dm*-*tra-2* ^*ts2*^ was shown to exhibit better fertility than *Dm*-*tra-2* ^*ts1*^ at permissive temperatures^1,18^, its mutation site was the initial choice for editing in *D. suzukii* for this study, though the *tra-2* ^*ts1*^ allele might also present advantages for application, or additional insights into function.

Similar to *Dm*-*tra-2* ^*ts2*^, XX chromosomal females homozygous for *Ds-tra-2* ^*ts2*^ reared at permissive temperatures of 16–20 °C resulted in development of normal fertile females, while rearing at a restrictive temperature of 26 °C resulted in a sterile, though incomplete pseudo-male phenotype. The lack of full male phenotypic differentiation, unlike XX; *Dm-tra-2* ^*ts2*^ pseudo-males reared at 29 °C, was most likely the result of rearing *D. suzukii* at a lower sub-optimal restrictive temperature, since this species fails to complete pupal development when reared above 26 °C. Thus, complete loss of Ds-TRA-2 function at 26 °C was unlikely, though distinct male morphological differentiation was apparent based on complete foreleg sex comb development, posterior dorsal tergite 6 pigmentation, and wing spot pigmentation when shifted to 29 °C in early adulthood. The genital apparatus structures were intersexual, but predominantly male-like, and similar to XX; *Dm-tra-2* ^*ts2*^ pseudo-males, the gonads of XX; *Ds-tra-2* ^*ts2*^ pseudo-males were dysmorphic, possibly intersexual, and did not produce motile sperm^1,19^. Conversely, XY; *Ds-tra-2* ^*ts2*^ males reared at 26 °C exhibited normal male external morphology and mated, but were also sterile with dysmorphic testes consistent with the *tra-2* null phenotype in *D. melanogaster* that is known to interfere with male germ-line development^12,13^. A notable difference between the *tra-2* ^*ts2*^ lines in the two species is that *Dm-tra-2* ^*ts2*^ XX females and XY males were highly sensitive to loss of Dm-TRA-2 activity, becoming sterile at 18 °C^18^, while *Ds-tra-2* ^*ts2*^ XX and XY individuals exhibited less sensitivity, becoming sterile at 22 °C and 26 °C, respectively.

While *D. suzukii* could not develop past late pupation at 29 °C, surviving adults at 26 °C or below could be reared at 29 °C, allowing the sex-specificity of downstream transcriptional products in adults to be assessed in response to Ds-TRA-2^ts2^ function at the optimal restrictive temperature. This allowed a more accurate comparison of TRA-2 function in *D. suzukii* and *D. melanogaster* and its downstream regulation of sexual differentiation. In *D. melanogaster*, functional TRA-2 acts in concert with the *transformer* gene product to facilitate the female-mode of *doublesex* alternative intron-splicing, resulting in DSX-Female function throughout adulthood^20^. The continuous need for control by the sex-determination gene pathway throughout female development was, indeed, first inferred by experiments with *Dm-tra-2* ^*ts2*^ that showed that female-specific *Yp* transcription and synthesis in adults could be up- and down-regulated by manipulating temperature-dependent Dm-TRA-2 function^5^. For Ds-TRA-2^ts2^, we showed that *Yp* gene expression could be similarly regulated by temperature such that XX; *Ds-tra-2* ^*ts2*^ pseudo-males reared at 16 °C and shifted to 29 °C at adult eclosion exhibited basal *Yp1* transcription levels, that could be transiently up-regulated to levels comparable to WT females after a shift to 16 °C, and subsequently down-regulated to basal levels by a shift to 29 °C. Together, these studies confirmed the expressivity and temperature-dependent conditional function of the gene-edited *Ds-tra-2* ^*ts2*^ mutation that also provided support for the continuous, and reversible, requirement for Ds-TRA-2 in both females and males for adult sexual differentiation and fertility throughout development.

Beyond providing a conditional system to better understand how sex-determination genes control sexual differentiation and behavior, the *Drosophila tra-2* ^*ts*^ studies also present a highly efficient strategy for generating a sterile male population for SIT^6^. For this, a *tra-2* ^*ts*^ mutant strain reared at permissive temperature would result in a normal breeding population of fertile males and females, whose chromosomal male and female progeny all develop as sterile phenotypic males at the restrictive temperature. Thus, ideally, a *tra-2* ^*ts*^ mutant strain could provide a complete sterile male population in response to a shift in temperature, and provide the significant efficiency of all zygotes being used for release, requiring a considerably smaller breeding population.

Current control of *D. suzukii* does not include SIT^21^, and the temperature-sensitive sexing system described is less than optimal for this species due to post-larval inviability at 29 °C required for complete XX female sex reversal. However, XY; *Ds-tra-2* ^*ts2*^ males are sterile but sexually active at ambient temperatures above 25 °C, and could be reared at lower temperatures for improved viability, yet sterilized when shifted to higher restrictive temperatures as pharate adults. These sterile males could directly compete with wild males in the field, though the ability of their sibling XX; *Ds-tra-2* ^*ts2*^ pseudo-males to provide mating competition remains uncertain. Regardless of their ability to mate with females in the field, chromosomal females that develop as phenotypic males or intersexuals would still preclude the need to eliminate or sterilize these individuals before release, unlike normal females, since they would not require sterilization, nor compete with females in the field for mating with released sterile males, or cause oviposition damage to host plants. Thus, the programmatic and cost advantages of *Ds-tra-2* ^*ts2*^-based sexing and sterility might still provide a level of efficiency that would support its incorporation into prospective SIT programs for *D. suzukii*. Nevertheless, the fertility and mating behavior of both XX and XY mutant adults would require further testing to ensure the efficacy of this system in response to temperature fluctuations under actual field conditions.

This demonstration of temperature-dependent sex reversal established by gene-editing in *D. suzukii* also supports the notion that this could be accomplished in other insect species having conserved *tra-2* cognate function and sequence identity. This is especially so for several tephritid fruit fly species, such as *Ceratitis capitata* (medfly), *Anastrepha ludens* (mexfly), and *A. suspensa* (caribfly), which have conserved sites for the TRA-2 Alanine and Proline peptides substituted in the *tra-2* ^*ts1*^ and *tra-2* ^*ts2*^ mutations, respectively^3,22^. Importantly, sex reversal of chromosomal females by RNAi-based knock-down of the *tra* and *tra-2* cognates, resulting in XX pseudo-males that copulate with wild females, has already been demonstrated for medfly and caribfly^22,23^. Given that these tropical insects thrive at ambient temperatures of 29 °C or above, it is likely that full male differentiation could be achieved in XX; *tra-2* ^*ts*^ individuals with the potential for successful mating with females in the field. However, the RNAi studies also showed that while *tra-2* knock-down in XX caribflies resulted in sterile pseudo-males, XY males remained fertile, and in medfly both XX pseudo-males and XY males remained fertile. Thus, for tephritid species *tra-2* ^*ts*^ males reared at restrictive temperatures might remain fertile, requiring an independent conditional system for male sterility, such as the tetracycline-dependent embryonic lethality system described for medfly and caribfly^24,25^.

In summary, proof of principle has been demonstrated in *D. suzukii* for the creation of a conditional temperature-sensitive allele by gene-editing that most likely can be extended to other temperature-sensitive missense mutations, and in a wide range of species known to have an orthologous gene^26^. That a very broad range of genes can be edited in this way in insects is supported by *D. melanogaster* mutational studies that showed that greater than 10% of X-linked lethal and semi-lethal mutations are temperature-sensitive^27^. For the *tra-2* gene in particular, that is conserved among several orders of insects, synthetic creation of temperature-sensitive mutations has significant potential for detailed comparative functional analyses and, for pest species, enhancing the efficacy of programs to control their population size. While this and other possible applications remain prospective, the ability to create conditional alleles in a large number of genes known to be important to many aspects of reproduction, development and behavior should have a highly significant impact on our understanding of genome function and evolution.

## Methods

All data generated or analysed during this study are included in this published article (and its Supplementary Information files). All sequence files are available from the GenBank database (accession numbers MF142759, MF142760, MF142761 and MF142762) after publishing of this work.

### Fly rearing, fertility and mating tests

A wild type strain of *D. suzukii* originally collected in Wimauma, Florida, was reared under standard laboratory conditions as described^28^. Gene-edited transgenic homozygous flies were reared at 16 °C ± 0.2 °C (EchoTherm™IN40 incubator, Torrey Pines Scientific, Inc.), and tested at indicated temperatures ( ± 0.2 °C) for temperature sensitivity after 24 h and 48 h of egg collection at 16 °C. Fertility tests were performed by mating each mutant male to five wild type virgin females (*n* ≥ 15) and each mutant female to three WT males (*n* ≥ 20) followed by egg collections for 12–15 days. Progeny were reared to adulthood and screened for red fluorescence. Mating tests were performed by combining previously unmated 3 d adult flies for observation of courtship behavior including body orientation, wing vibration, licking and copulation for 6 h at mid-day (1000–1600), and for 4 h beginning at dawn (approx. 0700) of the following day.

### Mutation generation via CRISPR/Cas9

The *Ds-tra-2* gene was identified by a Blast search using *Dm-tra-2* mRNA (GenBank: NM_057416.3) as a query, and was identified in contig1010 from the *D. suzukii* genome (GenBank: XM_017071951)^4^. The putative *Ds-tra-2* ^*ts2*^ mutation site was localized to nt 571 in the CDS (GenBank: MF142759) based on alignment with the *Dm-tra-2* ^*ts2*^ amino acid sequence^1,18^, and confirmed with primers AH1001/AH1002 by RT-PCR and sequencing. Oligonucleotide primers were designed with Geneious^®^ 8.0.5 (Biomatters Ltd.) software and listed in Supplementary Table S3 with annealing conditions. sgRNA targets were identified by a manual search for NGG proto-spacer adjacent motif (PAM) sequences. Two targets were identified having high GC content within the six PAM-proximal nucleotides (PAMPNs)^29^ and less similarity to other genomic sequences to improve efficiency and avoid off-target effects. sgRNAs were synthesized as previously described^30^ using primers AH992, AH1053 and AH1054 (Supplementary Table S3). Their specificities were confirmed by embryonic injections and Surveyor tests (Supplementary Fig. S2).

To create the donor construct, a 3.6 kb fragment from *D. suzukii* genomic DNA was cloned using Q5^®^ High-Fidelity DNA Polymerase (New England Biolabs) with primers AH1020/AH1021, which included two homologous arms of more than 1 kb upstream and downstream from the two sgRNA target sites. The *Ds-tra-2* ^*ts2*^ point mutation was introduced by primers AH1027/AH1028. To avoid cleavage of the donor construct and mutagenesis after integration by CRISPR/Cas9, two single-nucleotide synonymous substitutions (G > C for sgRNA1 site; G > A for sgRNA2 site) were introduced into the two sgRNA target site PAM sequences, respectively, since an intact PAM sequence is necessary for successful double strand breakage^31^. Homologous arm 1 (HA1) was assembled using the GeneArt^®^ Seamless Cloning and Assembly Kit (Thermo Fisher Scientific) from three fragments, which were amplified with primer pairs AH1026/AH1027, AH1028/AH1029, and AH1030/AH1031. HA2 was assembled from two fragments amplified with primer pairs AH1032/AH1033 and AH1034/AH1035 with the *Ds-tra-2* ^*ts2*^ mutation and PAM substitutions confirmed by sequencing. Next, a fragment containing the *IE1hr5-DsRed/*SV40 marker gene was amplified from the *piggyBac* transformation vector plasmid, pB{*IE1hr5-DsRed/*SV40} with primers AH1044/AH1045, and the complete HA1 and HA2 fragments were amplified using primer pairs AH1026/AH1043 and AH1035/AH1046. The DsRed marker, HA1 and HA2 were then assembled with the GeneArt^®^ Kit and confirmed by sequencing.

The *D. suzukii Ds-ligase4* gene (GenBank: XM_017079597)^4^ was identified by Blastn alignment similarity to *Dm-ligase4* from *D. melanogaster* (GenBank: NM_132679.3; protein alignment of the conceptual translation of these sequences shown in Supplementary Fig. S4). The online SnapDragon tool (http://www.flyrnai.org/snapdragon) was used to design a 421 bp dsRNA fragment whose sequence was confirmed after PCR amplification with primers AH1003/AH1004 (GenBank: MF142760). The dsRNA was then synthesized as described previously^22,32^.

### Embryo micro-injection

Pre-blastoderm embryos were injected with a mixture of 400 ng/µl Cas9 protein (PNA Bio Inc), 75 ng/µl sgRNA1, 75 ng/µl sgRNA2, 100 ng/µl donor plasmid and 350 ng/µl *Ds-ligase4* dsRNA using *D. suzukii* germ-line transformation injection methods^28^. Putative G1 transformant adults were selected by DsRed epifluorescence (TxRed filter: ex: 560/40; em: 610 LP; Chroma) that were backcrossed to wild type with G2 homozygous lines established by successive inbreeding, selection by marker fluorescence intensity, and verification by outcrosses to wild type. Genomic DNA of homozygous flies was extracted with ZR Genomic DNA™-Tissue MicroPrep (Zymo Research) with the edited regions amplified with primer pairs AH1093/AH986 and AH987/AH1094 and sequenced for molecular confirmation.

### Cuticle preparation

Flies were dissected in phosphate-buffered saline (PBS) solution with forelegs, abdominal and terminal cuticles incubated in 10% NaOH at 80 °C for 30 minutes to remove soft tissue, rinsed with PBS and mounted in Acrytol Mounting Media (Electron Microscopy Sciences) for imaging.

### Real-time qPCR

Total RNA was isolated with the ZR Tissue & Insect RNA MicroPrep™ kit (Zymo Research), followed by cDNA synthesis from 1 μg RNA using the iScript™ cDNA Synthesis Kit (Bio-Rad). The *Ds-Yp1* (GenBank: XM_017081211) and *Ds-His3* (GenBank: XM_017082962) genes were identified by a Blastn search of the *D. suzukii* genome^4^ with their *D. melanogaster* cognates (GenBank: NM_078548.3 for *Yp1* and Flybase: CG33866-RA for *His3*). Primer pairs AH1133/AH1134 and AH1135/AH1136 were used to amplify their partial CDS (GenBank: MF142761 for *Ds-Yp1*, and MF142762 for *Ds-His3*) whose sequence identities were verified by Blastx and ClustalW^33^ alignments (Supplementary Figs S1 and S5). Primers AH1139/AH1140 and AH1137/AH1138 were designed to compare the expression levels of *Ds-Yp1* to the internal control *Ds-His3* gene, respectively, using a CFX96 Touch™ Real-Time time PCR Detection System (Bio-Rad). 100 ng cDNA was added into iQ™ SYBR^®^ Green Supermix (Bio-Rad). The 2^−∆∆Ct^ method was used with three biological replicates for each sample at each time point^34^, with all data normalized to the relative *Yp1*/*His3* expression of sample M36f1 at 3 d–29 °C (mean = 0.2), and the Student *t-test* was performed to compare differences.

## Electronic supplementary material

Supplementary Information

Video S1

Video S2
