# Supplementary material for: Temperature-dependent sex-reversal by a transformer-2 gene-edited mutation in the spotted wing drosophila, Drosophila suzukii
Source: Sci Rep. 2017 Sep 28;7:12363. doi: 10.1038/s41598-017-12405-4 (PMC5620132; doi:10.1038/s41598-017-12405-4)
Supplement: Supplementary file 1 — Supplementary Information [file 41598_2017_12405_MOESM1_ESM.pdf]

Temperature-dependent sex-reversal by a *transformer-2* gene-edited mutation in the spotted wing drosophila, *Drosophila suzukii*

Jianwei Li* and Alfred M. Handler

Supplementary Information

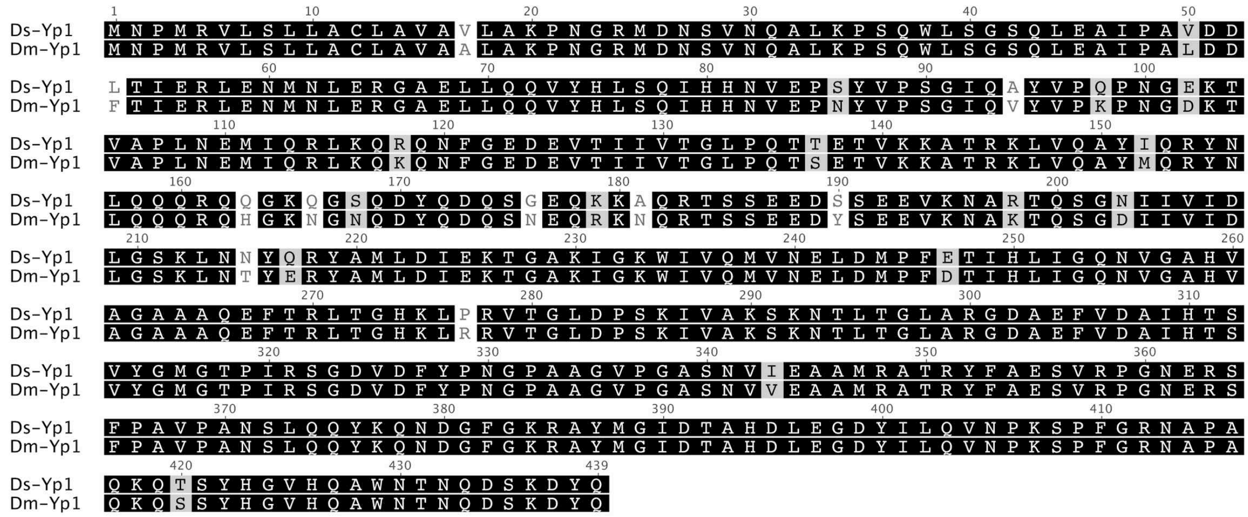

Figure S1. ClustalW multiple sequence alignment of the *D. suzukii* Ds-Yp1 and *D. melanogaster* Dm-Yp1 proteins.

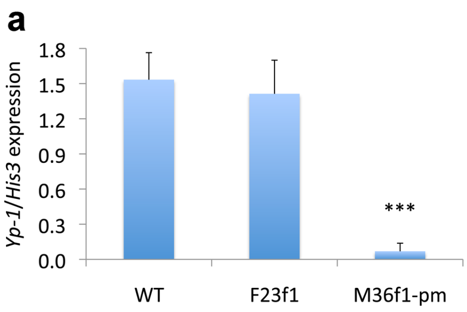

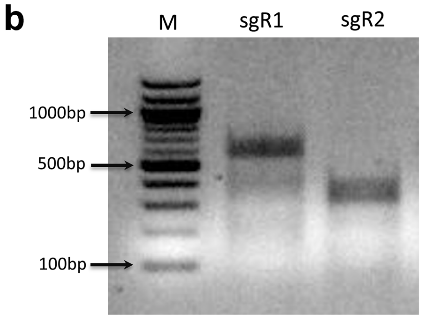

Figure S2. Comparison of *Ds-Yp1* expression and confirmation of Cas9/sgRNAs cutting specificity.
a) Comparison of *Ds-Yp1* expression in 7 day old WT and control females, and M36f1 pseudo-males at 26°C. Abbreviations: WT, wild type females; F23f1, control line females; M36f1-pm, XX; *Ds-tra-2*^{ts2} pseudo-males. F23f1 control females are *Ds-tra-2*$^{+}$, but express the *IE1hr5-DsRed* marker gene from an independent pB{*IE1hr5-DsRed/*SV40} transformation. The asterisks (*) above M36f1 mark the student *t-test* results compared to WT: ***: $P < 0.001$ (0.0002). F23f1 is not significantly different from wild type ($P = 0.36$). b) Confirmation of Cas9/sgRNAs cutting specificity. For sgRNA1 (sgR1), the amplified fragment was 597 bp using primers AH1082/1083, while the Surveyor test generated fragments of ~333 bp and ~264 bp due to a relatively low number of mutations generated by Cas9/sgRNA1. For sgRNA2 (sgR2), primers AH1084/1052 amplified a fragment of 332 bp, while the Surveyor test generated fragments of ~243 bp and ~96 bp due to a relatively low number of mutations generated by Cas9/sgRNA2.

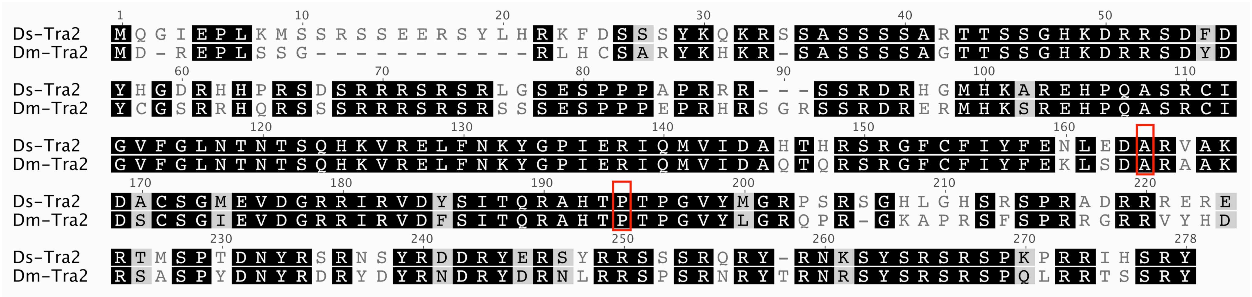

Figure S3. ClustalW multiple sequence alignment of the Ds-TRA-2 and Dm-TRA-2 proteins. Red boxes mark the position of the Ds-TRA-2^{ts1} and Ds-TRA-2^{ts2} mutations.

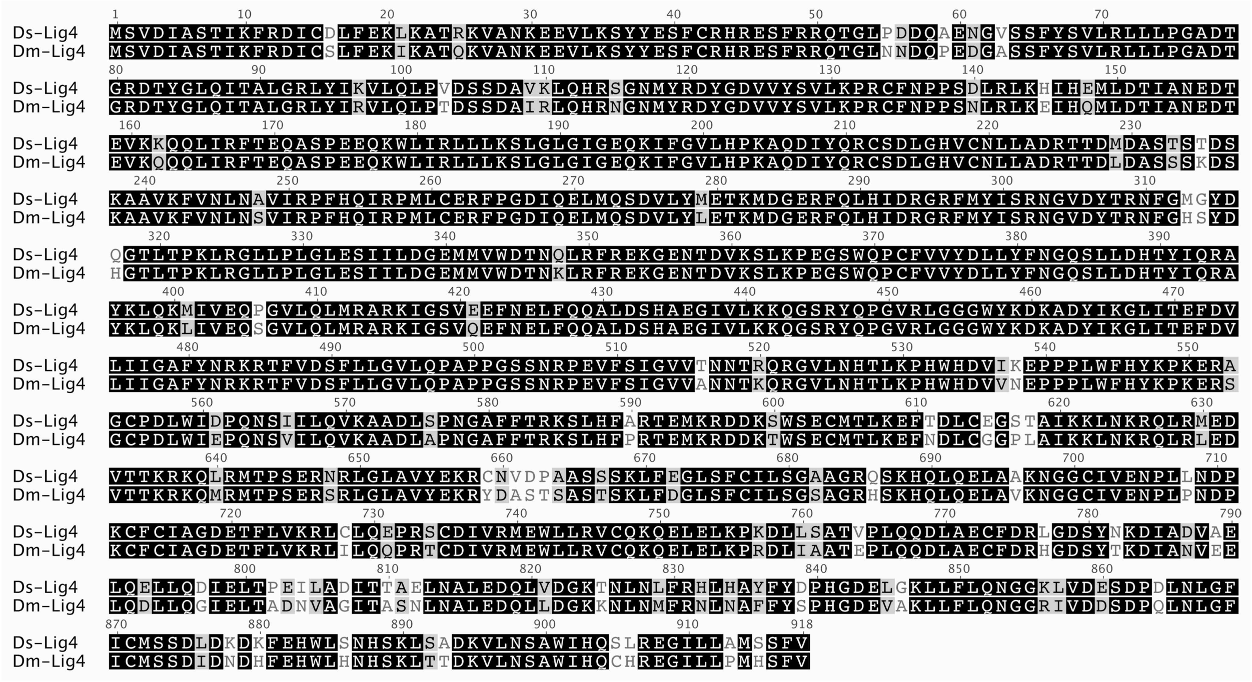

Figure S4. ClustalW multiple sequence alignment of the Ds-Lig4 and Dm-Lig4 proteins.

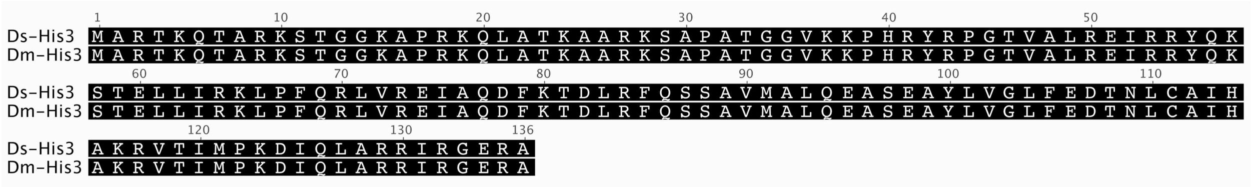

Figure S5. ClustalW multiple sequence alignment of the Ds-His3 and Dm-His3 proteins.

Table S1. Phenotype and fertility of the *Ds-tra-2^{ts2}* line under temperature shifts to improve viability

Group	Temperature shifts[a]	XY Males			XX Females		
		No.	Fertility (%)	Immotile sperm[b]	No.	Intersex[c]	Fertility (%)
1)	22°_AE > 26°_3d > 26°	20	10	3/5	31	+	0
2)	22°_11d > 26°_AE_3d > 26°	33	5	3/5	34	+	0
3)	22°_9d > 26°_AE > 29°_3d > 26°	95	0	4/5	35	++	0
4)	22°_9d > 29°_AE_3d > 26°	89	0	4/5	32	++	0

[a] Eggs were collected at 16°C or 20°C for a 24-48h period and reared for indicated number of days (d) or to adult eclosion (AE) at 22°C; subsequent shifts in temperature and time periods indicated by (>); the time to AE for each group: 1) 12d, 2) 1d, 3) 2d, and 4) 2d; fertility was tested at the last temperature. The viability in all groups was between 80% and 90%, which was estimated based on the progeny from same number of WT adults. Wild type flies were phenotypically normal and fertile under all four temperature shifts ($n \geq 20$).

[b] males with only immotile sperm/ males dissected (see Video S1 and S2 for comparison to wild type sperm number and motility).

[c] (+) denotes sex combs and abnormal genitalia; and (++) denotes sex combs, abnormal genitalia and wing spots.

Table S2. Relative *Ds-Yp1* transcript expression to the internal control *Ds-His3* under different temperature conditions

Temperature conditions [a]	Time points	Relative *Ds-Yp1* expression (mean ± SD)			*t-test* of M36f1 to WT-f (*P* value)
		WT-f	M36f1	WT-m	
16°>16°>16°C	3d-16°C	2.7 ± 3.6	4.3 ± 7.2	-	0.8015
	6d-16°C	37.8 ± 26.6	32.8 ± 12.6	-	0.7814
	9d-16°C	29.7 ± 14.7	15.6 ± 6.7	-	0.3079
29°>29°>29°C	3d-29°C	26.9 ± 2.8	1.0 ± 0.99	0	0.0003
	6d-29°C	6.2 ± 0.9	0.44 ± 0.22	0	0.0002
	9d-29°C	3.8 ± 0.3	0.017± 0.006	0	0.0001
29°>16°>16°C	3d-29°C	26.9 ± 2.8	1.0 ± 0.99	-	0.0003
	6d-16°C	16.9 ± 1.9	9.4 ± 4.9	-	0.1413
	9d-16°C	14.2 ± 7.5	10.8 ± 1.9	-	0.4957
29°>16°>29°C	3d-29°C	26.9 ± 2.8	1.0 ± 0.99	-	0.0003
	6d-16°C	16.9 ± 1.9	9.4 ± 4.9	-	0.1413
	9d-29°C	8.5 ± 5.2	0.18 ± 0.11	-	0.0246

[a] Fresh XX; *Ds-tra2*^{ts2} and wild type flies reared at 16°C were constantly kept at 16°C or 29°C for 9 d; shifted to 29°C for 3 d, then to 16°C for another 3 d (indicated by (>)), then kept at 16°C or shifted to 29°C for additional 3 d.

Table S3. Oligonucleotide primer sequences (5’ to 3’ orientation) and their annealing temperature

Primer	Ta[a] (°C)	Sequence
AH1001	72	GAACACCAACACCTCGCAGCAC
AH1002	72	CGTGAGCGGCTGTAGCTTTTGTT
AH992	60	AAAAGCACCGACTCGGTGCCACTTTTTCAAGTTGATAACGGACTAGCCTTATTTTAACTTGCTATTTCTAGC
AH1053	60	GAAATTAATACGACTCACTATAGGCGCGAACCTTGTGCTGCGGTTTTAGAGCTAGAAATAGC
AH1054	60	GAAATTAATACGACTCACTATAGGTAGGCCAGTTCCAGGCTCGTTTTAGAGCTAGAAATAGC
AH1020	70	GGCCGAGTAAAGGTTAAGGTGTCT
AH1021	70	CCGCTGATGGAGGTGTATAGGTTAC
AH1026	80	ATTCGAGCTCGGTACGGCCGAGTAAAGGTTAAGGTGTCT
AH1027	80	CCTTGTGCTGCGACGTGTTGGTGTTCAGACC
AH1028	82	ACACCAACACGTCGCAGCACAAGGTTCGCG
AH1029	82	CCGGAGTAGAAGTGTGCGCGCGTTGGGTTATGGAGTA
AH1030	77	CAACGCGCGCACACTTCTACTCCGGGTGTCTATATG
AH1031	77	CCAAGCTTGCATGCCAGACTCAACTTTCTGTACAATTTCA
AH1032	70	ATTCGAGCTCGGTACGTTGAGTCTTTTGTTCTTTATTTAT
AH1033	70	GCATGTGATAAATCAGAGCCTGGAACT
AH1034	71	TCCAGGCTCTGATTTATCACATGCATATCC
AH1035	71	CCAAGCTTGCATGCCGCTGATGGAGGTGTATAGGTTA
AH1044	72	TGAGTCTGCTTTAAGATACATTGATGAGTTTGGACA
AH1045	72	AAAGACTCAACCGAATTCCTGCAGCCCG
AH1043	69	TCTTAAAGCAGACTCAACTTTCTGTACAATTTCAATT
AH1046	69	AATTCGGTTGAGTCTTTTGTTCTTTATTTATTGAATTGGT
AH1003	73	TCTGGCCGAGTGCTTTGACCGTC
AH1004	73	GCCCTCCCGAAGCGATTGATGA
AH1093	71	TGCATCACCTCGAACAGCAGAG
AH986	71	CCTTCGCCTGGGACATCCT
AH987	71	GATGGTGTAGTCCTCGTTGTGGG
AH1094	71	CTTGGTGGAGTTGCTGGAGTTCTT
AH1133	69	ACAACTCCGTCAACCAGGC
AH1134	69	GCACCGACATTCTGGCCAATC
AH1135	69	CCACGCAAACAACTGGCTACT
AH1136	69	CAGAGCCATAACCGCCGAG
AH1139	56	CTGCAGCAAGTCTACCACCTG
AH1140	56	GCGCTGGATCATCTCGTTCAG
AH1137	56	GAACGGTTGCCCTGCGTG
AH1138	56	AGCTCTGGAATCGCAGGTCAG
AH1082	68	GGACTTCGATTACCATGGCGAT
AH1083	68	GAACGATGGGTCTGTATGGCA
AH1084	69	CTTGCCGACGATGAAGTGGAG
AH1052	69	CTCCAACGAACTTACCCAATCCCT

[a] Ta = annealing temperature.

Video S1. Immotile sperm from XY; *Ds-tra-2^{ts2}* chromosomal males at 3 d after adult eclosion. This is a time-lapse of a 21 second period compressed into one second.

Video S2. Motile sperm from XY WT males at 3 d after adult eclosion. This is a time-lapse of a 21 second period compressed into one second.
